# Progress of Rapid Detection Technology for Aquatic Microorganisms: A Comprehensive Review

**DOI:** 10.3390/microorganisms14040939

**Published:** 2026-04-21

**Authors:** Qin Liu, Zhuangzhuang Qiu, Mengli Yao, Boyan Jiao, Yu Zhou, Chenghua Li, Haipeng Liu, Lusheng Xin

**Affiliations:** 1 School of Public Health, Jining Medical University, Jining 272002, China; 2 College of Agricultural Engineering, Guangxi Vocational University of Agriculture, Nanning 530007, China; liuq@gxnzd.edu.cn; 3 Department of Laboratory, Jining Center for Disease Control and Prevention, Jining 272000, China; 4 College of Marine Science, Ningbo University, Ningbo 315832, China; 5 College of Ocean and Earth Sciences, Xiamen University, Xiamen 361005, China

**Keywords:** aquatic environment, microorganisms, detection method, nucleic acid level

## Abstract

Microbial contamination in aquatic environments poses severe threats to aquaculture sustainability, ecological balance and public health. Traditional culture-based detection methods, while standardized, are time-consuming and labor-intensive, often failing to meet the urgent need for rapid on-site monitoring required to prevent disease outbreaks and manage water quality effectively. By integrating latest research advances (2020–2025), this study reviews advances in rapid detection technologies for aquatic microorganisms, including the evolution of nucleic acid amplification strategies, with a focused comparison of the analytical sensitivity and field deployability of quantitative polymerase chain reaction (qPCR) and mainstream isothermal amplification techniques (loop-mediated isothermal amplification, LAMP; recombinase polymerase amplification, RPA). Furthermore, this study reports on the emergence of Clustered Regularly Interspaced Short Palindromic Repeat (CRISPR)-associated protein (Cas) systems as next-generation diagnostic tools, highlighting their integration with microfluidic Lab-on-a-Chip (LOC) platforms to achieve attomolar sensitivity. We also consider the application of portable nanopore sequencing for real-time pathogen identification and the growing role of Artificial Intelligence (AI) in analyzing complex diagnostic datasets. Advanced molecular methods have achieved significant reductions in time consumption—from days to less than one hour—while challenges regarding sample preparation and environmental matrix inhibition remain. The future of aquatic monitoring lies in integrated, automated systems that combine the specificity of CRISPR-Cas diagnostics with the connectivity of IoT-enabled biosensors. Comparative analysis indicates that isothermal amplification methods (LAMP, RPA) coupled with CRISPR-Cas systems offer the optimal balance of sensitivity, speed, and field deployability for point-of-care aquaculture diagnostics, while qPCR/dPCR remain indispensable for quantitative regulatory applications. We propose a structured technology selection framework to guide researchers and practitioners in choosing appropriate detection modalities based on specific sensitivity, cost, throughput, and deployment requirements.

## 1. Introduction

### 1.1. The Critical Importance of Aquatic Microbial Monitoring

Aquatic pathogenic microorganisms destabilize aquatic economies and jeopardize human health. Some of these pathogens are a leading cause of gastrointestinal disorders, diarrhea, and systemic infections in human globally [1]. The World Health Organization (WHO) and other international organizations have recognized that contaminated water sources contribute significantly to the global burden of disease, with waterborne outbreaks resulting in substantial economic and social losses [2]. In parallel, the aquaculture sector, which is an important source of high-quality protein, faces constant threats from infectious diseases, the spread of antimicrobials, antibiotic-resistant bacteria and genes in aquatic environments [3]. As farming practices intensify to meet global food demands, the prevalence and impact of viral and bacterial pathogens in aquaculture, as well as associated antimicrobial resistance, have escalated, necessitating robust biosecurity and water quality management measures [4]. Consequently, the surveillance of microbial water quality has received much attention in recent years due to its pivotal role in preventing disease outbreaks and maintaining the integrity of aquatic ecosystems [5,6].

**Figure 1:**
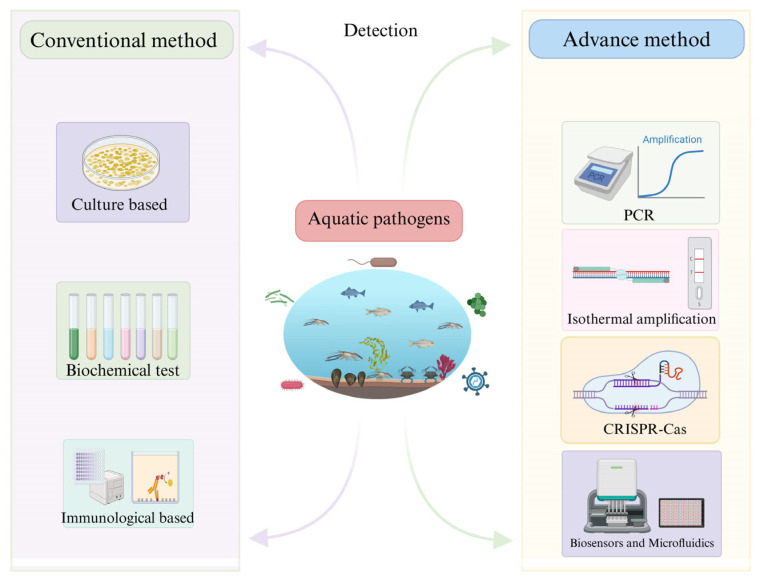
The development of Aquatic Pathogen Detection Technology.

The scope of aquatic microbial monitoring extends beyond direct microbial pathogens to include the surveillance of antimicrobial resistance (AMR) genes and the assessment of microbial community shifts that indicate environmental degradation. Microbial pollutants threaten the balance of aquatic ecosystems, compromising biodiversity and the resilience of natural water bodies [7,8]. Additionally, the economic implications of pathogen outbreaks in aquaculture are profound. Viral and bacterial disease such as Tilapia lake virus (TiLV) or *Vibrio* species can rapidly decimate farmed stocks, causing multi-billion-dollar global losses and threatening farmers’ livelihoods [9,10]. Therefore, the development of detection technologies that are not only accurate but also rapid and field-deployable has become a central priority in aquatic microbial monitoring (Figure 1) [5,11].

### 1.2. Limitations of Traditional Detection Paradigms

Historically, microbial assessment has commonly relied on culture-based enumeration of fecal indicator bacteria and pathogens, which remains the regulatory gold standard due to its standardization, low cost, and direct confirmation of viable cells [12,13]. However, these approaches are labor-intensive and time-consuming, typically requiring 18–72 h, and up to several days for some pathogens, before results are available, making them poorly suited for early warning and rapid response frameworks in aquaculture surveillance [14,15]. In addition, culture methods may underestimate risk by failing to detect pathogens that enter a viable but non-culturable (VBNC) state under environmental stress, in which cells retain membrane integrity, metabolic activity and virulence, but lose the ability to grow on standard media, leading to false negative results and underestimation of public health risks [12,13,15,16], and they generally cannot detect viruses or slow-growing protozoa without specialized host cell lines, further increasing the technical complexity and cost [17].

Traditional immunological methods such as the enzyme-linked immunosorbent assay (ELISA) provide a faster alternative to culture by detecting pathogen-specific antigens, with high analytical specificity and suitability for high-throughput screening [18]. However, in environmental waters where pathogens are typically present at very low concentrations, conventional ELISA formats often require prior enrichment and may fail to reach the sensitivity needed for large-volume monitoring [19]. Furthermore, specific antibodies are unavailable for most aquatic pathogens, and generating such antibodies is labor-intensive, time-consuming and costly. Assay performance is also hampered by matrix interference and poor antibody stability in field settings, particularly resource-limited ones [5,20]. These drawbacks have driven extensive research into nucleic acid-targeted molecular detection strategies (PCR, qPCR, isothermal amplification, next-generation sequencing), which offer far greater sensitivity and speed for aquatic microbial monitoring than phenotype or antigen-based approaches [21,22,23,24].

### 1.3. The Evolution of Nucleic Acid Diagnostic Technologies

The advent of PCR in the 1980s revolutionized aquatic microbiology by enabling the exponential amplification of trace amounts of nucleic acids from water and host tissues, independent of culturability [5,25]. Over subsequent decades, conventional PCR and its quantitative variant, real-time PCR (qPCR), have become cornerstone molecular tools in aquaculture diagnostics and environmental surveillance owing to their rapid turnaround, high analytical sensitivity and specificity [26,27]. By directly targeting pathogen DNA or RNA, qPCR permits detection and quantification of non-culturable or difficult-to-culture agents, including viruses, protozoa and VBNC bacteria, and has been successfully applied for early warning of viral and parasitic infections at very low copy numbers in aquaculture [28,29,30]. Recent advances in multiplex and high-throughput qPCR enable simultaneous detection of multiple pathogens in a single reaction, with a limit of detection typically 10–100 times lower than conventional PCR, greatly facilitating the diagnosis of co-infections and large-scale surveillance of bacterial, viral and parasitic diseases in aquaculture systems [31,32,33,34].

However, despite their widespread adoption, PCR-based assays generally rely on sophisticated thermocyclers, costly reagents, and trained personnel, which confines most applications to centralized laboratories [35,36]. This centralization introduces a logistical bottleneck, as samples must be transported from the field to the laboratory and queued for processing, reintroducing delays that can offset the intrinsic speed of the amplification reaction itself [37,38]. Moreover, environmental and wastewater samples often contain PCR inhibitors (e.g., humic substances, organic compounds, heavy metals) that disrupt nucleic acid extraction, template integrity and DNA polymerase activity, requiring extensive purification to prevent false negatives [39,40]. To overcome these issues, recent research has focused on isothermal amplification technologies—notably loop-mediated isothermal amplification (LAMP) and recombinase polymerase amplification (RPA)—which enable nucleic acid amplification at a constant temperature without thermal cycling. This allows for simpler, low-power devices that are more suitable for point-of-care and on-site testing [36,41].

More recently, the integration of Clustered Regularly Interspaced Short Palindromic Repeat (CRISPR) technology with nucleic acid amplification has emerged as a disruptive innovation [42]. By leveraging the RNA-guided, sequence-specific cleavage activities of CRISPR-associated (Cas) proteins together with the exponential signal generation of amplification reactions, these hybrid platforms achieve markedly enhanced analytical sensitivity and specificity, particularly in point-of-care settings [43,44]. CRISPR-Cas systems that employ Cas12 or Cas13 effectors exploit their programmability and multiple-turnover trans-cleavage activity to reach near single-molecule detection limits while discriminating single-nucleotide variants, thereby addressing false positive signals that commonly arise from non-specific amplification in conventional assays [45,46]. Concurrently, advances in microfluidics have enabled the miniaturization of these complex biochemical workflows onto “Lab-on-a-Chip” (LOC) devices, promoting the development of automated, sample-to-answer platforms [47].

### 1.4. Aims and Structure of This Review

Numerous high-quality systematic reviews have advanced the global understanding of waterborne and aquatic pathogen detection technologies, each delivering unique, specialized contributions to the field. A series of focused reviews have addressed individual core detection technologies including PCR, biosensors, and next-generation sequencing (NGS) in isolation, providing detailed, targeted insights into the technical evolution and application of each methodology, and establishing robust foundational references for specialized research in this domain. Meanwhile, several recent comprehensive reviews have offered valuable overviews of broader waterborne pathogen detection technologies: Feleni et al. surveyed the latest developments in waterborne pathogen detection technologies with a core focus on the innovation and application of biosensors and molecular methods [6], while Oon et al. provided a broad, holistic overview of mainstream waterborne pathogen detection technologies alongside an in-depth analysis of key challenges in their practical implementation [5].

Building on these robust foundational contributions from the existing literature, this review delivers a targeted, systematic synthesis of rapid detection technologies for aquatic microorganisms, with a specific focus on advancements reported from 2020 to 2025, tailored to the practical application context of aquaculture systems. The primary aims of this review are as follows: to systematically compare the efficacy, analytical sensitivity, and field-applicability of emerging molecular and biosensor-based detection technologies; to establish a set of clear criteria for selecting appropriate detection methods based on target pathogen characteristics and on-site environmental settings; and to describe the integration of artificial intelligence (AI) and microfluidic technologies as key future drivers of intelligent water quality monitoring in aquaculture.

The remainder of this review is organized as follows. Section 2 summarizes the latest advances in nucleic acid amplification strategies, including polymerase chain reaction (PCR), quantitative PCR (qPCR), digital PCR (dPCR), and mainstream isothermal amplification technologies. Section 3 outlines recent progress in CRISPR-based diagnostic systems for aquatic pathogen detection, with a dedicated integrated comparison of CRISPR-Cas12a and Cas13a platforms in the aquaculture context. Section 4 reviews biosensors and microfluidic lab-on-a-chip platforms developed for automated, field-deployable detection. Section 5 discusses advances in NGS-based detection approaches and artificial intelligence/machine learning (AI/ML)-assisted data analytics for aquatic diagnostics. Section 6 provides a systematic cross-technology comparison, presents a structured technology selection framework (Table 3) aligned with specific aquaculture use scenarios, and highlights current technical limitations and future development opportunities in the field. Finally, Section 7 summarizes the core conclusions and outlook of the review.

### 1.5. Literature Search and Study Selection Strategy

A structured literature search was conducted across Web of Science, PubMed, Scopus, and Google Scholar databases from January 2020 to June 2025. The primary search terms included combinations of (“aquatic” OR “aquaculture” OR “water” OR “fish” OR “shrimp” OR “shellfish”) AND (“pathogen detection” OR “microbial detection” OR “molecular diagnostics”) AND (“PCR” OR “qPCR” OR “dPCR” OR “LAMP” OR “RPA” OR “isothermal amplification” OR “CRISPR” OR “Cas12” OR “Cas13” OR “biosensor” OR “microfluidic” OR “lab-on-a-chip” OR “next-generation sequencing” OR “metagenomics” OR “artificial intelligence” OR “machine learning”). Secondary searches were performed for specific topics including antimicrobial resistance detection, IoT-enabled monitoring, and smartphone-based diagnostics.

Inclusion criteria required that studies (1) describe detection methods applied to aquatic microorganisms, aquaculture pathogens, or waterborne pathogens; (2) report original data on assay performance (sensitivity, specificity, limit of detection, or turnaround time); and (3) be published in English in peer-reviewed journals. Review articles were included when they provided systematic synthesis relevant to specific technology categories. Exclusion criteria removed studies focused exclusively on clinical human diagnostics without aquatic or environmental application, non-peer-reviewed conference abstracts, and studies published before 2020 unless they represented foundational methodological references. Foundational references (pre-2020) were retained when necessary to describe core principles and mechanisms of established technologies (e.g., PCR, LAMP, CRISPR mechanisms).

The initial search yielded approximately 1200 records. After removing duplicates and screening titles and abstracts, 380 articles were assessed for full-text eligibility. A total of 171 references were included in the final review, with priority given to studies demonstrating field validation, novel platform integration, or comparative performance data. The reference lists of included articles and relevant review papers were also searched to identify additional studies not captured by database queries.

## 2. Nucleic Acid Amplification Strategies: From PCR to Isothermal Systems

The detection of specific genomic segments remains the most reliable method for identifying aquatic microorganisms. This section evaluates the current state of PCR technologies and the shift towards isothermal amplification for field applications, detailing the mechanisms, advantages, and limitations of each approach.

### 2.1. Polymerase Chain Reaction (PCR) and Its Variants

PCR-based methods have long been the cornerstone of molecular diagnostics. Standard end-point PCR, while useful for qualitative detection, has largely been superseded by quantitative PCR (qPCR) for environmental monitoring due to the latter’s ability to provide real-time quantification of pathogen load [48,49].

#### 2.1.1. Quantitative PCR (qPCR)

qPCR employs fluorescent probes or intercalating dyes to monitor DNA amplification in real time. Recent developments have emphasized multiplex qPCR, which enables the simultaneous detection of multiple pathogens within a single reaction, thereby increasing throughput and reducing cost. In aquaculture, multiplex and high-throughput qPCR assays have been established to identify co-infections by diverse viral and bacterial agents in a single reaction, greatly facilitating disease surveillance and management [32,50,51,52]. qPCR exhibits excellent analytical sensitivity, with optimized assays frequently achieving limits of detection on the order of 10 or even fewer copies of the target gene per reaction [53,54,55,56].

The mechanism of quantitative PCR (qPCR) relies on sequence-specific primers combined with either a fluorogenic probe (e.g., TaqMan hydrolysis probes) or a double-stranded DNA-binding dye (e.g., SYBR Green) to monitor amplification in real time. During thermal cycling, the target DNA is exponentially amplified and the fluorescence signal increases accordingly, generating a characteristic amplification curve whose shape depends on the monitoring chemistry and PCR efficiency [57,58,59]. The quantification cycle (Cq), defined as the cycle number at which the fluorescence signal first exceeds a background threshold, is inversely related to the logarithm of the initial target copy number and can be converted into absolute or relative concentrations using a calibrated standard curve [60].

This quantitative capability is particularly important in water quality and recreational water studies, where qPCR-derived concentrations of pathogen genes or microbial source tracking markers are used as inputs to quantitative microbial risk assessment (QMRA) models [61,62,63,64]. Numerous investigations in recreational lakes, marine bathing waters, karst aquifers and groundwater-fed drinking water systems have shown that health risk depends strongly on pathogen or marker concentration, not just on their presence/absence [65,66]. For example, qPCR-based measurements of *Salmonella*, *Mycobacterium avium*, *Pseudomonas aeruginosa* and human-associated *Bacteroides* (HF183) have been linked to estimated infection probabilities that exceed or fall below regulatory risk benchmarks depending on gene copy numbers per 100 mL of water [61,62,63,65,66]. Thus, quantifying target abundance via Cq-based qPCR is crucial for distinguishing low-level contamination from concentrations that represent a meaningful public health threat in aquatic environments.

However, qPCR remains highly susceptible to inhibition by complex environmental matrices. Co-extracted substances in aquatic samples, such as humic and fulvic acids in freshwater, organic matter and metal ions in sewage, or elevated ionic strength and salts in marine systems, can suppress DNA polymerase or reverse transcriptase activity, alter Mg^2+^ availability, bind nucleic acids, or dampen fluorescence signals, thereby delaying Cq values and even causing false negatives [67,68,69,70,71]. Numerous studies on environmental waters and eDNA have shown that such inhibition is common and, if left unaddressed, can lead to substantial underestimation of target concentrations and high proportions of missed detections in large-volume surveillance samples [67,69,72,73,74]. Thus, robust upstream workflows for sample concentration and nucleic acid extraction are essential for reliable qPCR analysis of aquatic environmental samples. These include optimized filtration, bead-beating as needed, use of inhibitor-resistant mastermixes, targeted inhibitor removal (e.g., DAX-8 for humic substances), and amplification controls to identify and mitigate inhibition [67].

In addition, the requirement for precise thermal cycling between denaturation, annealing, and extension temperatures (typically around 95 °C, 60 °C, and 72 °C) has historically necessitated benchtop instruments with power-intensive Peltier elements, restricting the portability of conventional qPCR platforms for true field deployment [75]. Recent advances in low-power thermal cyclers using polymer-based microchips, thin-film platinum heaters, fan-based cooling, and microcontroller-driven control electronics demonstrate that accurate PCR amplification can be achieved with compact, battery-operated devices, substantially reducing energy consumption while maintaining temperature accuracy [76,77]. Nonetheless, the combination of strict thermal requirements, power management, and environmental robustness still represents a major engineering challenge for fully field-deployable qPCR systems.

#### 2.1.2. Digital PCR (dPCR)

Digital PCR (dPCR), and specifically droplet digital PCR (ddPCR), represents a third-generation PCR technology that enables absolute quantification of nucleic acids without standard curves by partitioning a sample into thousands to millions of independent reactions [78]. In ddPCR, the reaction mix is emulsified into tens of thousands of nanoliter droplets that act as individual microreactors. Amplification occurs only in droplets with at least one target copy, and target concentration is calculated from the ratio of positive to negative droplets via Poisson statistics [79]. This partitioning confers several analytical advantages over qPCR. First, dPCR provides direct, calibration-free quantification with well-defined confidence intervals and excellent inter-laboratory comparability [60]. Second, by isolating template molecules into small volumes and reading endpoint fluorescence, ddPCR shows enhanced tolerance to partial PCR inhibition, which is especially beneficial for turbid or chemically complex water, wastewater, and eDNA samples that contain humic substances, salts, or other inhibitors [25,80]. In such matrices, ddPCR frequently achieves higher detection rates and more stable copy number estimates than qPCR, particularly at very low pathogen or eDNA concentrations near the limit of detection [81,82]. These characteristics make dPCR a powerful tool for environmental surveillance, including quantification of fecal indicators, microbial source-tracking markers, cyanobacteria, and waterborne pathogens in ambient waters and wastewater [83,84].

Nevertheless, dPCR also has its limitations. Current platforms typically have higher capital and per-sample costs, lower throughput, and longer turnaround times than qPCR, and still require careful assay optimization and standardized data analysis to avoid biases from dead volume, partition loss, or saturated reactions [60,80,85]. As a result, dPCR is often adopted as a complementary, higher-precision method for targeted applications rather than as a universal replacement for qPCR in routine monitoring.

### 2.2. Isothermal Amplification Technologies

**Figure 2:**
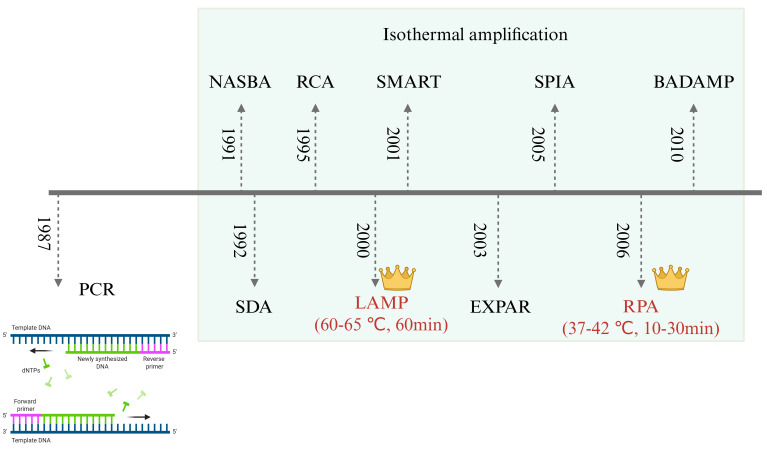
Development of Isothermal Amplification Technology.

To overcome the reliance of traditional PCR on thermal cycling equipment, isothermal amplification technologies have emerged, enabling efficient nucleic acid amplification at a constant temperature, which is suitable for rapid on-site detection (Figure 2). These methods simplify instrumentation requirements, reduce energy consumption, and significantly advance the development of point-of-care diagnostics (Table 1). Nucleic Acid Sequence-Based Amplification (NASBA) is tailored for RNA amplification, with high performance in RNA virus detection; Strand Displacement Amplification (SDA) achieves DNA amplification without thermal denaturation; Rolling Circle Amplification (RCA) produces highly sensitive long repetitive sequences using circular DNA as a template; Signal-Mediated Amplification of RNA Technology (SMART) relies on signal amplification instead of target replication. Due to its operational simplicity and visualizable results, Loop-Mediated Isothermal Amplification (LAMP) has become one of the most widely used methods in pathogen diagnosis. Single Primer Isothermal Amplification (SPIA) uses chimeric primers for rapid detection; Exponential Amplification Reaction (EXPAR) drives exponential amplification of short nucleic acids (e.g., microRNA); Recombinase Polymerase Amplification (RPA) is characterized by rapid reactions and compatible portable equipment; Dual Priming Isothermal Amplification (DAMP) achieves high sensitivity with low background via multiplex primer design. These technologies have markedly improved the accessibility and practicality of on-site diagnostics by simplifying workflows, cutting costs and boosting detection efficiency. RPA and LAMP are the most widely used in recent research, and thus form the primary focus of the following review.

#### 2.2.1. Loop-Mediated Isothermal Amplification (LAMP)

Loop-mediated isothermal amplification (LAMP) has emerged as a widely used alternative to PCR for field and point-of-care diagnostics. It employs 4–6 primers targeting 6–8 distinct regions of the target DNA and a strand-displacing DNA polymerase, most commonly Bst, enabling highly efficient amplification at a constant temperature of 60–65 °C and yielding up to ~10^9^ copies of the target within 1 h [41,86]. The core mechanism involves coordinated action of inner and outer primers to generate dumbbell and stem-loop structures in the early phase of the reaction; these structures then serve as self-priming templates, driving rapid accumulation of concatemeric products containing inverted repeats of the target sequence [87].

Recent developments have greatly expanded the application of loop-mediated isothermal amplification (LAMP) and reverse-transcription LAMP (RT-LAMP) for pathogen detection in aquaculture and aquatic environments, where they are now widely used for the rapid diagnosis of bacterial, viral and microsporidian pathogens of fish and shellfish [88,89,90]. For example, closed-tube colorimetric LAMP assays have been established for the shrimp microsporidian *Enterocytozoon hepatopenaei* (EHP), enabling visual detection from hepatopancreas, feces, pond water and even soil within ~40–45 min using hydroxynaphthol blue or phenol red, with diagnostic sensitivity and specificity approaching 100% under farm-level conditions [91]. Similar visual LAMP–HNB formats have been reported for the fish pathogen *Aeromonas salmonicida*, providing rapid naked-eye detection from infected fish samples with sensitivity surpassing conventional PCR and strong agreement with bacteriological diagnosis [92]. Colorimetric or fluorometric LAMP assays have also been developed for other aquaculture-relevant microbes, including *Rahnella aquatilis* and *Yersinia ruckeri* in fish, achieving limits of detection down to a few tens of copies per microliter or <1 cell per microliter in filtered water, and total assay times of <1 h, thus supporting routine surveillance based on environmental DNA from aquaculture waters [89,93].

LAMP has rapidly evolved from a laboratory technique into a core platform for field-deployable aquatic microbiology diagnostics, particularly suited to aquaculture where early, on-site detection of pathogens is crucial for disease control and food safety [89,92,94].

Compared with PCR, LAMP’s isothermal operation and tolerance to crude samples enable portable formats such as microfluidic chips, smartphone-based readers and handheld detectors, which are increasingly being tailored for water and farm-side testing [35,95]. For example, LAMP microfluidic and polydimethylsiloxane (PDMS) chip systems now allow for multiplex detection of common shrimp pathogens (DIV1, WSSV, EHP) with limits of detection around 10 copies/reaction and total assay times of 35–40 min while maintaining qPCR-comparable performance in clinical samples [88,95]. Similar thinking has been applied to fish and waterborne bacteria: colorimetric LAMP assays targeting virulence or marker genes enable naked-eye detection of *Aeromonas veronii*, *Morganella morganii* and *Yersinia ruckeri* in aquaculture water around 10 CFU/mL within 20–30 min, with diagnostic agreement close to PCR or qPCR but without thermocyclers [89,96,97]. LAMP has also been expanded to parasites and oomycetes, such as *Dactylogyrus* spp. and *Saprolegnia* spp., where assays coupled to lateral-flow dipsticks or simple heating blocks reach fg-level DNA sensitivity and detect single zoospores, supporting proactive surveillance in freshwater systems [94,98]. More advanced probe-based and hybrid formats further address traditional LAMP limitations in multiplexing and non-specific amplification: probe-LAMP and LAMP-PfAgo duplex systems, as well as tRCA-LAMP and CuNCs-LAMP, improve specificity, quantification and resistance to background interference when detecting multiple foodborne or aquatic pathogens in seafood and environmental samples [99,100,101,102]. Overall, the trajectory of LAMP in aquatic microorganisms is moving from single-target, lab-bound assays toward integrated, multiplex, and user-friendly point-of-care testing (POCT) platforms, but challenges remain in standardizing sample preparation, minimizing false positives in complex matrices, and validating assays across diverse real-world farm environments [35,99,103].

A critical assessment of the LAMP studies reviewed here reveals important limitations in validation scope. The majority of reported limits of detection (LODs) (typically 1–100 copies/reaction) were determined using serially diluted plasmids or purified genomic DNA, with fewer studies demonstrating comparable performance in complex environmental matrices such as pond water, sediment, or crude tissue homogenates. Furthermore, the reported turnaround times (20–45 min) generally refer to the amplification step alone and do not include sample collection, nucleic acid extraction, or result interpretation, which can add 30–60 min to the total workflow. Only a minority of studies have conducted multi-site field validation or compared performance across diverse water types (freshwater, brackish, marine), limiting the generalizability of reported performance metrics.

#### 2.2.2. Recombinase Polymerase Amplification (RPA)

RPA is a rapid isothermal nucleic acid amplification technology that operates at a constant low temperature (typically 25–42 °C) and amplifies DNA or RNA within 10–30 min, making it highly attractive for point-of-need detection of aquatic microorganisms [104,105,106]. RPA has emerged as a powerful point-of-care nucleic acid platform for aquatic microbial detection, particularly suited to farm- and pond-side diagnosis where infrastructure and technical expertise are limited. Conceptually, RPA reaction at 37–42 °C with lyophilized reagents and minimal instrumentation positions it as a practical alternative to PCR for real-time health monitoring in aquaculture system [107,108,109]. In fish and shrimp disease control, RPA and RT-RPA combined with lateral flow dipsticks (RPA-LFD) or fluorescence readout now support rapid detection of a wide spectrum of pathogens, including major viruses (CyHV-3, LYCIV, LMBRaV, TiLV, ISKNV, MRV, SDDV, DIV1) and shrimp pathogens such as AHPND-causing *Vibrio* spp. and *Enterocytozoon hepatopenaei* [110,111,112,113,114,115,116]. For example, real-time RPA on a centrifugal microfluidic chip enables parallel detection of five key shrimp pathogens at 39 °C in 20 min with LO around 10 copies/µL and clinical sensitivity/specificity of 96.4%/100% relative to PCR, illustrating how RPA can be embedded in highly automated platforms for routine pond monitoring [110]. At the other end of the device spectrum, simple RPA-LFD assays for *Flavobacterium columnare* or DIV1 operate at ~37–40 °C, deliver visible results in ~30 min, and reach sensitivities of 0.4 CFU or about 10 copies/µL, comparable to or better than conventional PCR while tolerating crude fish or shrimp tissues [115,117]. A clear trend is the coupling of RPA with next-generation signal amplification technologies—CRISPR/Cas12a or Argonaute—to push sensitivity down to single-copy or sub-copy levels and to enable multiplexed, visual detection in the field for viruses such as SDDV, LMBV, ISKNV and MRV or for EHP in shrimp supply chains [113,118,119,120].

Critical analysis shows RPA is advancing from proof-of-concept to integrated surveillance tools, yet its performance relies on rigorous primer/probe optimization to suppress non-specific amplification and standardized sample preparation workflows compatible with complex farm and environmental matrices [107,116]. RPA platforms exhibit impressive analytical performance, with sensitivity often below 10 copies/µL and turnaround times of 10–30 min, but these metrics are mostly obtained under controlled laboratory conditions using purified templates or spiked samples. Few studies validate RPA with naturally infected clinical samples or field-collected environmental specimens, and the reported turnaround times typically exclude sample preparation steps. Furthermore, the stability of lyophilized RPA reagents under tropical field conditions, the main hub of global aquaculture production, has not been systematically assessed over extended storage periods.

### 2.3. Comparative Assessment of NAATs

Before comparing these platforms, it is important to distinguish between analytical sensitivity and diagnostic sensitivity/specificity. Analytical sensitivity refers to the lowest concentration of target that can be reliably detected by the assay, typically expressed as LOD. Diagnostic sensitivity refers to the proportion of truly infected/positive samples correctly identified by the assay, expressed as a percentage. Similarly, analytical specificity (the ability of the assay to detect only the intended target without cross-reactivity) differs from diagnostic specificity (the proportion of truly negative samples correctly identified). Many studies reviewed here report primarily analytical sensitivity (LOD using purified targets), while diagnostic sensitivity and specificity—which require validation against a reference standard using clinical or environmental samples—are reported less consistently. This distinction is critical for translating laboratory-optimized assays to field applications, where matrix effects, co-infecting organisms, and varying pathogen loads can substantially reduce diagnostic performance relative to analytical benchmarks.

**Figure 3:**
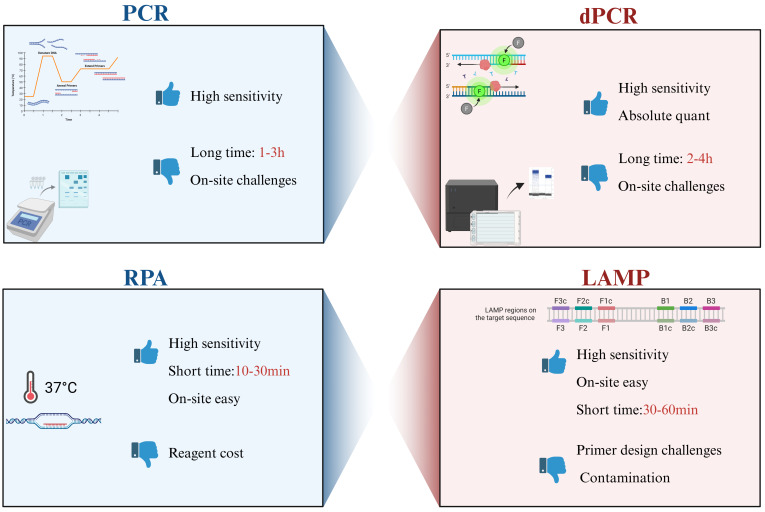
Comparison of PCR, dPCR, RPA, and LAMP Technologies.

The choice between PCR, LAMP, and RPA depends on the specific application scenario (Figure 3). PCR and qPCR remain unmatched for high-throughput, centralized laboratory testing where quantification accuracy is paramount. In contrast, LAMP and RPA are superior for decentralized, point-of-care testing where speed and simplicity are critical. The integration of these isothermal methods with microfluidic chips (discussed in Section 4) further enhances their potential by automating the complex sample handling steps.

**Table 1:** The key differences between these technologies, highlighting the trade-offs between sensitivity, speed, cost, and field availability.

Feature	PCR/qPCR	Digital PCR (dPCR)	LAMP	RPA
Amplification Mechanism	Thermal cycling	Partitioning + Thermal cycling	Isothermal (60–65 °C)	Isothermal (37–42 °C)
Sample Type	Purified DNA/RNA; limited for crude samples	Purified DNA/RNA; improved tolerance for crude samples	Crude/processed samples (e.g., swabs, tissue homogenates)	Crude/processed samples (e.g., swabs, blood, environmental eluates)
Extraction Requirements	High-purity nucleic acid; mandatory extraction	High-purity nucleic acid; minimal extraction needed	Minimal extraction; compatible with direct lysis	Minimal extraction; compatible with direct lysis
Validation Status	Well-validated; gold standard for diagnostics	Highly validated; niche clinical/research use	Widely validated; point-of-care (POC) applications	Strongly validated; emerging POC/field use
Multiplexing Capacity	Moderate (≤4-plex via probes; limited by channel number)	Low (constrained by partitioning/volume)	High (up to 8-plex; limited by primer compatibility)	Moderate (≤3-plex; challenges with probe design)
Quantitative Capability	Semi-quantification (standard curve required)	Absolute quantification (no standard curve needed)	Semi-quantitative; semi-quantitative endpoint	Semi-quantitative; semi-quantitative endpoint
Robustness Under Field Conditions	Low (requires power, thermal cycler, controlled temp)	Low (requires specialized instrumentation)	High (simple heating, portable devices)	Very High (no thermal control, battery-operated compatible)
Estimated Cost per Test	Low (instrument + reagents; ~$1/test)	Very High (instrument + reagents; ~$10–$50/test)	Low (simple reagents; ~$1/test)	Low–Moderate (specialized reagents; ~$5–$10/test)
Reagent Stability	Cold-chain required (2–8 °C); short shelf-life	Cold-chain required; ultra-stable reagents	Ambient/stable refrigeration; long shelf-life	Ambient/stable refrigeration; long shelf-life
Contamination Risk	High (amplicon carryover; aerosol risk)	Very High (digital partitioning reduces but does not eliminate risk)	Very High (high amplicon yield; aerosol risk)	High (high amplicon yield; non-specific background noise risk)
Technology Readiness Level (TRL)	TRL 9 (market-ready, clinical routine)	TRL 8–9 (clinical adoption; niche use)	TRL 9 (commercialized, POC widespread)	TRL 8–9 (commercialized, expanding field use)
Regulatory Status	FDA/CE marked for most diagnostics	FDA/CE approved for limited applications	FDA/CE marked for multiple POC assays	FDA/CE authorized for select assays

## 3. CRISPR-Cas Systems: The Next Frontier in Diagnostic Specificity

While isothermal amplification methods such as LAMP and RPA have significantly improved the speed and portability of aquatic pathogen detection, they remain susceptible to non-specific amplification that can compromise diagnostic accuracy. The integration of CRISPR-Cas systems addresses this limitation by providing an additional layer of sequence-specific verification, offering ultra-specific, field-deployable tools for pathogen surveillance in aquaculture and aquatic environments.

### 3.1. Mechanism of CRISPR-Based Detection

CRISPR-Cas systems are adaptive immune systems in bacteria and archaea that use a Cas nuclease guided by a programmable CRISPR RNA (crRNA) to recognize and cleave complementary nucleic acid targets. Among the diverse CRISPR-Cas types, Type V (Cas12a/b) and Type VI (Cas13) have been most widely adapted for diagnostic applications due to their unique collateral cleavage activity [121,122,123]. Upon binding to the target sequence, these nucleases undergo a conformational change and exhibit trans-cleavage of nearby reporter single-stranded nucleic acids, releasing fluorescent, colorimetric, or electrochemical signals with high sequence specificity [122,124,125]. Unlike Cas9, which is primarily used for gene editing and performs only cis-cleavage of the target DNA, Cas12 and Cas13 effectors exhibit multiple-turnover trans-cleavage activity, effectively amplifying the detection signal. Target recognition typically requires a protospacer adjacent motif (PAM) near the target site, which constrains detectable sequences but also contributes to specificity. Coupling CRISPR readout with isothermal amplification enables attomolar-to-femtomolar sensitivity at constant temperatures, making these platforms well suited for on-site applications [121,122,123,126].

CRISPR-Cas diagnostics offer several strategic advantages for aquatic monitoring. First, multiple studies have demonstrated detection limits comparable to qPCR while using simple heating blocks or portable readers, bridging the gap between centralized laboratories and pond-side decision-making [113,127,128,129,130,131,132]. Second, the same platform can be rapidly reprogrammed to detect diverse targets—viruses, bacteria, toxin genes, and resistance determinants—simply by changing the crRNA and primers. Third, integration with microfluidic chips and electrochemical sensors enables closed-tube, low-contamination platforms that minimize operator skill requirements [113,127,130,132,133].

### 3.2. Applications in Aquatic Pathogen Detection

**Figure 4:**
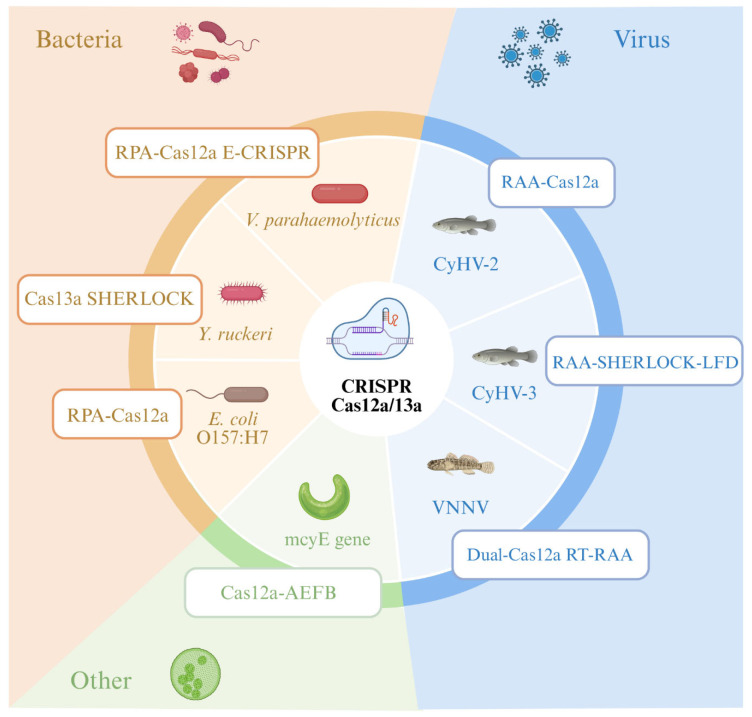
Applications of CRISPR-Cas12a/13 Detection Technology.

CRISPR-Cas12a and Cas13 systems combined with recombinase-based amplification have been demonstrated for rapid diagnosis of major aquatic pathogens (Figure 4). Table 2 summarizes representative platforms developed for fish, shellfish, and environmental samples.

Analysis of the representative CRISPR-Cas platforms developed for aquatic pathogen detection (Table 2) reveals several overarching trends. First, Cas12a-based systems dominate the current landscape, accounting for the majority of reported assays, largely because their dsDNA trans-cleavage activity is directly compatible with amplicon products from RPA/RAA preamplification that is the most commonly used isothermal method in these studies. In contrast, Cas13a systems, which target RNA via ssRNA collateral cleavage, have been primarily deployed for RNA virus detection (e.g., NNV) and biofilm-associated surveillance (e.g., *Yersinia ruckeri* detection via SHERLOCK [128]), but require an additional transcription step that complicates one-pot integration. Second, a clear design trajectory is evident from early two-step protocols (separate amplification and CRISPR detection) toward one-pot formats that simplify field workflows, though sensitivity trade-offs persist due to enzyme compatibility constraints. Third, critical evaluation reveals significant validation gaps: the majority of platforms have been validated only with spiked samples or limited clinical panels (n < 50), with few studies reporting multi-site field trials across diverse water types and species. Sample preparation, particularly nucleic acid extraction from turbid, inhibitor-rich aquaculture water, remains the most consistent bottleneck, with only a minority of platforms achieving extraction-free or fully integrated workflows.

Among viral pathogen applications, the coupling of RAA/RPA with Cas12a has enabled rapid detection of major aquaculture viruses including CyHV-2 [125], CyHV-3 [132], and NNV [133], with LODs ranging from 0.5 to 100 copies per reaction and turnaround times of 30–60 min. Notably, some systems have incorporated Fe-based flocculation for non-destructive monitoring from aquaculture water [125], representing progress toward integrated sample-to-answer workflows. For bacterial pathogens, electrochemical CRISPR biosensors (e.g., RPA-Cas12a for *Vibrio parahaemolyticus* [126]) and SHERLOCK-based systems (e.g., for *Y. ruckeri* biofilm surveillance [128]) demonstrate the versatility of CRISPR platforms across different target types and sample matrices. Beyond pathogen detection, CRISPR systems have been extended to environmental monitoring of cyanotoxin genes [124] and antimicrobial resistance determinants [130], broadening their utility for comprehensive aquaculture biosecurity.

### 3.3. Limitations and Future Directions

Despite the transformative potential of CRISPR-Cas diagnostics, several challenges remain before these systems can achieve true “sample-in, answer-out” operation in aquaculture settings. Most assays still require nucleic acid extraction or heat/chemical pretreatment, particularly for bacteria, biofilms, and sediment samples, which prevents fully automated workflows from raw environmental samples [136,137,138]. While microfluidic CRISPR chips have integrated magnetic extraction and lysis protocols, many remain semi-manual bench-top devices rather than fully autonomous field sensors [136,139]. Additionally, *Cas* proteins, isothermal amplification kits, and labeled reporters typically cost more than conventional PCR reagents, and dependence on proprietary formulations raises supply chain concerns [137,138]. Scaling to multi-pathogen panels requires orthogonal Cas effectors or microfluidic partitioning to control cross-talk, and robust regulatory adoption demands multicenter validation against qPCR and culture methods across diverse species and water types [140,141].

The choice between two-step and one-pot reaction formats represents a critical design trade-off. Two-step protocols retain maximal sensitivity and single nucleotide polymorphism (SNP) discrimination but introduce transfer steps and contamination risks, whereas one-pot formats simplify workflows but must reconcile the temperature and buffer requirements of multiple enzymes [138,142]. Recent advances in thermostable Cas12a/Cas12b orthologs with robust activity at 55–65 °C now support LAMP-CRISPR one-pot assays, substantially advancing field-ready diagnostics [137,139,143]. Amplification-free approaches utilizing CasΦ and collateral cleavage enhancement are also emerging as promising strategies to eliminate amplification entirely [143,144]. Future developments will likely prioritize amplification-free sensing enhanced by advanced optics or electrochemistry, fully integrated microfluidic devices requiring only sample loading, and networked low-cost sensor arrays for real-time pathogen and AMR tracking across aquaculture facilities [125,140,144]. The following section examines how microfluidic Lab-on-a-Chip platforms and biosensor technologies address these integration challenges.

## 4. Biosensors and Microfluidics: Towards Automated Monitoring

The molecular amplification and CRISPR-Cas technologies discussed earlier deliver exceptional sensitivity and specificity, yet their widespread on-site use for aquatic monitoring requires integration into automated, user-friendly platforms. Microfluidic lab-on-a-chip systems, biosensor-based devices and compact optical readers are pivotal to translating benchtop assays into field-deployable tools [145,146]. These platforms enable rapid, low-cost and operator-independent analysis by miniaturizing fluid handling, integrating sample preparation and coupling with simple visual, electrochemical or smartphone-assisted readouts [147]. This section reviews recent advances in microfluidic, biosensor and optical/smartphone-enabled systems tailored for aquatic pathogen detection.

### 4.1. Microfluidic Lab-on-a-Chip (LOC) Platforms

Microfluidics manipulates nanoliter-to-microliter volumes in channels typically below 1 mm in dimension, enabling precise control of fluid flow and reaction conditions. LOC systems reduce reagent consumption, accelerate mass and heat transfer, and confine amplified products within sealed chambers, thereby minimizing contamination risk [1,148]. Importantly, they permit sequential integration of lysis, nucleic acid extraction, amplification, and detection into a single device, supporting sample-to-answer workflows for aquatic pathogen surveillance [1,148,149].

Environmental water and aquaculture samples contain particulate debris, organic matter, and PCR/LAMP inhibitors that compromise downstream assays. Recent LOC designs therefore incorporate on-chip filtration, pathogen concentration, and magnetic bead-based nucleic acid extraction to process raw wastewater or surface water without extensive off-chip pretreatment [1,149,150]. Effective world-to-chip interfaces, encompassing inlet design, preconcentration modules and inhibitor removal, are critical for translating microfluidic assays from model buffers to complex environmental matrices [1,149].

Several recent studies illustrate the potential of integrated LOC platforms for aquatic pathogen detection. Huang and Jiang [149] developed a centrifugal microfluidic disk integrating virus concentration, purification, and droplet digital RT-LAMP for quantifying pepper mild mottle virus in raw sewage; the platform achieved quantitative detection over 6.0 × 10^5^–2.1 × 10^9^ copies/mL within 90 min, with performance comparable to conventional extraction methods. Jena et al. [150] created an origami paper-based microfluidic device combining thermal lysis, cationic microgel-enhanced DNA concentration, LAMP, and colorimetric readout, achieving limits of detection of 10 CFU/mL for *Escherichia coli* and *Salmonella* spp. in drinking water within 75 min. Geissler et al. [148] reported a thermoplastic centrifugal LOC automating thermal lysis, PCR, hybridization and colorimetric detection of foodborne viruses, discriminating *hepatitis* A virus at 350 copies/μL through a fully automated 24-step workflow.

Overall, microfluidic LOC platforms have progressed from proof-of-concept devices to increasingly robust sample-to-answer systems capable of handling complex aquatic matrices. Remaining challenges include reducing device and reader costs, standardizing performance across diverse water types, and conducting large-scale field validations.

### 4.2. Biosensor Technologies

#### 4.2.1. Electrochemical Biosensors

**Figure 5:**
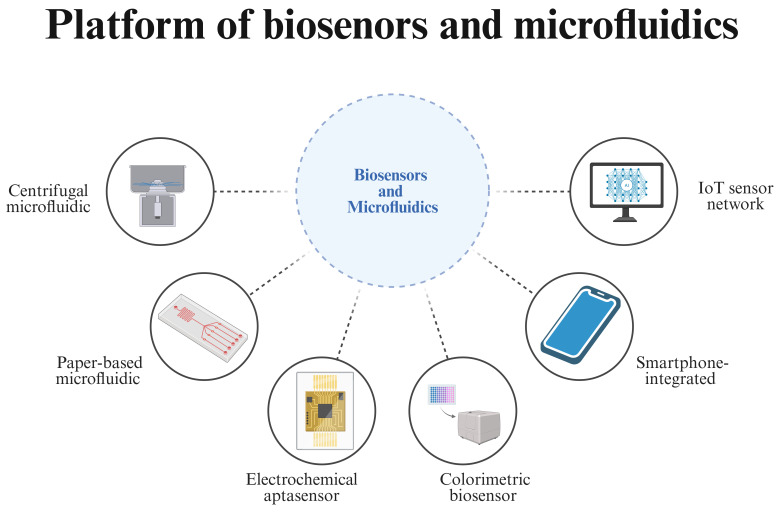
Biosensor and microfluidic platforms for aquatic pathogen detection.

Electrochemical biosensors convert biorecognition events into measurable electrical signals via amperometric, impedimetric, or voltammetric readouts. Their high sensitivity, compatibility with miniaturized electrodes, and low power requirements make them attractive for continuous aquatic monitoring (Figure 5) [151,152,153].

Pham et al. [152] reported a label-free electrochemical aptasensor for the freshwater cyanobacterium *Anabaena* sp. ULC602 using square-wave voltammetry at a gold electrode. Aptamer conformational changes upon target binding decreased peak current, enabling selective detection from OD_750_ 0.3 to 1.2 within one hour in freshwater samples. Chen et al. [154] designed a differential pulse voltammetric aptasensor for the shellfish toxin okadaic acid using thionine-functionalized g-C_3_N_4_ nanosheets, achieving a remarkably low LOD of 0.080 pg/mL (10^−15^ M) with successful quantification in contaminated shellfish extracts within 60 min.

#### 4.2.2. Aptamer-Based Biosensors

Aptamers are single-stranded DNA or RNA oligonucleotides that fold into defined structures to bind targets with high affinity and specificity. Selected in vitro through SELEX, aptamers offer superior stability, batch-to-batch reproducibility, and regeneration capability compared with antibodies, making them well suited for aquatic sensing applications [151,153,155,156].

Salleh et al. [157] developed a gold nanoparticle-based cortisol aptasensor for non-invasive fish stress monitoring in recirculating aquaculture systems. MgCl_2_-induced gold nanoparticle (AuNP) aggregation modulated by cortisol binding enabled colorimetric detection down to 100 pM within 35 min, with good agreement to HPLC in tank water samples. Rhouati and Zourob [153] reported a multiplexed electrochemical aptasensor for simultaneous detection of five cyanotoxins (microcystin-LR, cylindrospermopsin, anatoxin-a, saxitoxin, okadaic acid) in freshwater, achieving LODs of 0.0033–0.0053 nM with 90–109% recoveries in spiked tap water.

#### 4.2.3. Hybrid and Integrated Approaches

Recent biosensor designs combine aptamers, CRISPR systems, and nanozymes to enhance sensitivity and functional versatility. Xu et al. [128] established an RPA-mediated electrochemical CRISPR/Cas12a biosensor for *Vibrio parahaemolyticus* in fish, where Cas12a trans-cleavage of electrode-bound ssDNA probes yielded an LOD of 32 CFU/mL within 50 min. Liu et al. [132] created a dual-modal aptamer/CRISPR-Cas12a platform for simultaneous detection of azithromycin and the macrolide resistance gene ermB in aquatic environments, achieving picomolar LODs (2.34–9.17 pM) and >90% antibiotic degradation within 60 min. These integrated architectures demonstrate the potential for combining molecular recognition, CRISPR signal amplification, and catalytic remediation in future autonomous water quality management systems.

Collectively, the biosensor platforms reviewed in this section illustrate a convergence toward integrated architectures that combine molecular recognition, signal amplification, and catalytic functionality. However, a critical comparison reveals that most electrochemical and aptamer-based biosensors have been validated primarily in buffer systems or spiked samples, with limited demonstration in authentic environmental matrices. The long-term stability of aptamer-functionalized sensor surfaces under continuous exposure to aquaculture water, which contains biofilms, organic fouling agents, and variable ionic strength, remains insufficiently characterized. Furthermore, while LOD values in the picomolar to nanomolar range are frequently reported, these analytical benchmarks may not directly translate to diagnostic performance in complex field samples.

### 4.3. Optical, Smartphone, and IoT-Enabled Detection Systems

Optical biosensors for aquatic pathogens exploit colorimetric readouts for naked-eye screening and fluorescent modes for higher sensitivity. Zhang et al. [158] developed a printed nanoarray colorimetric biosensor that concentrates bacteria via evaporative capillarity, achieving an LOD of 10 CFU/mL with rapid visualization of bacterial load in water within minutes. Such optical formats are attractive for equipment-free on-site assessment of water quality.

Smartphone integration leverages the phone camera as an imaging detector, on-board processing for quantitative analysis, and wireless connectivity for data sharing. Yin et al. [159] assembled a 3D-printed microchannel fluorescent sensor coupled to a smartphone, using aptamer probes and magnetic separation to simultaneously detect *Staphylococcus aureus*, *Escherichia coli*, and *Pseudomonas aeruginosa* at 10 CFU/mL within 40 min. Pan et al. [160] reported a melamine-foam colorimetric biosensor for *E. coli* O157:H7, where smartphone-assisted imaging improved the LOD to 5 CFU/mL in agricultural water within 56 min. These studies demonstrate how smartphone-based analytics can convert qualitative optical changes into quantitative, sharable surveillance data.

IoT-enabled systems extend single-point measurements into continuous, distributed monitoring networks. Typical architectures combine in situ sensors with microcontrollers, wireless modules, and cloud dashboards for real-time visualization [161,162,163,164]. While most current deployments focus on physicochemical parameters (pH, temperature, dissolved oxygen) rather than pathogen-specific sensing, they demonstrate reliable long-term operation in aquaculture systems and provide a pathway for embedding molecular biosensors into smart aquaculture infrastructures [161,162]. Key challenges include sensor fouling, power management, and calibration drift. To facilitate platform selection, Table 3 compares the key performance characteristics of the technologies discussed in this section.

**Table 3:** Comparison of biosensor and microfluidic platforms for aquatic pathogen detection.

Feature	Centrifugal Microfluidic	Paper-Based Microfluidic	Electrochemical Aptasensor	Colorimetric Biosensor	Smartphone-Integrated	IoT Sensor Network
Detection Method	Integrated LAMP/RT-LAMP/PCR	LAMP + colorimetric	Impedimetric/Voltammetric	AuNP aggregation/Nanozyme	Camera imaging + app analysis	Continuous multi-parameter
Typical LOD	10^2^–10^3^ copies/mL	10 CFU/mL	0.001–100 nM (toxins); 10–100 CFU/mL (bacteria)	10–100 CFU/mL; pM (small molecules)	5–10 CFU/mL	Variable (primarily physicochemical)
Time to Result	60–90 min	15–30 min	30–60 min	20–60 min	40–60 min	Real-time
Portability	Moderate	Very high	High	Very high	Very high	Fixed installation
Sample Type	Purified nucleic acid, crude water homogenate, tissue lysate	Crude water, swabs, tissue homogenate, shellfish homogenate	Crude water, serum, tissue homogenate, environmental eluates	Crude water, swabs, food homogenate, environmental samples	Crude water, swabs, homogenate, field-collected samples	In situ water matrix (no sampling required)
Extraction Requirements	Mandatory (integrated on-chip nucleic acid extraction)	Minimal (compatible with direct lysis, no full purification)	Minimal (direct sample loading, no extraction for most matrices)	Minimal (no extraction required for visual detection)	Minimal (direct loading, compatible with crude samples)	None (in situ monitoring, no sample processing)
Validation Status	Well-validated for aquatic pathogens; standardized lab protocols	Widely validated for field use; POCT-focused assays	Highly validated for toxin/bacteria detection; research-to-commercial transition	Strongly validated for rapid screening; widely used in field trials	Moderately validated; emerging for aquatic diagnostics	Well validated for environmental monitoring; industrial-scale deployment
Multiplexing Capacity	Moderate (2–4-plex, limited by amplification chemistry)	Low (1–2-plex, constrained by colorimetric readout)	High (up to 6-plex, via multi-electrode arrays)	Low (1–2-plex, limited by color differentiation)	Moderate (2–3-plex, via app-based signal analysis)	Very High (multi-parameter, simultaneous physicochemical + biological monitoring)
Quantitative Capability	Absolute/relative quantitative (via qPCR/dPCR integration)	Semi-quantitative (endpoint color intensity)	High quantitative (calibrated electrochemical signal)	Semi-quantitative (visual/colorimetric intensity)	Semi-quantitative (app-calibrated image analysis)	Continuous quantitative (real-time sensor calibration)
Robustness Under Field Conditions	Moderate (requires controlled power, temperature, and sample handling)	Very High (simple operation, resistant to environmental interference)	High (portable instrumentation, stable in field conditions)	Very High (no power/instrumentation, field-ready)	Very High (battery-powered, field-deployable)	High (fixed installation, weather-resistant, low maintenance)
Estimated Cost per Test	High ($10–$30 per test, including chip and reagents)	Low ($1–$5 per test, low-cost paper substrates)	Moderate ($3–$10 per test, electrode and aptamer costs)	Low ($0.5–$3 per test, low-cost nanomaterials)	Low ($1–$5 per test, leveraging existing smartphone hardware)	High (infrastructure cost, $1000–$10,000 per node; low per-test operational cost)
Reagent Stability	Cold-chain required (2–8 °C for amplification enzymes)	Ambient-stable (lyophilized reagents, long shelf-life)	Ambient-stable (aptamer-modified electrodes, refrigerated storage optional)	Ambient-stable (AuNP/nanozyme reagents, room-temperature storage)	Ambient-stable (lyophilized assay reagents, field-compatible)	Not applicable (sensor hardware, no consumable reagents)
Contamination Risk	High (amplicon carryover, aerosol risk from nucleic acid amplification)	Very High (high LAMP amplicon yield, aerosol contamination risk)	Low (no amplification, direct detection, minimal carryover risk)	Low (no amplification, direct visual detection, no carryover)	Moderate (amplicon-based assays carry contamination risk; non-amplification assays are low-risk)	Not applicable (no sample processing, no contamination risk)
TRL/Readiness Level	TRL 8–9 (commercialized for clinical/lab use, emerging for aquatic POCT)	TRL 9 (fully commercialized, widely deployed for field POCT)	TRL 7–8 (proven in field trials, moving to commercialization)	TRL 8–9 (commercialized for rapid screening, field-ready)	TRL 7–8 (proven in field trials, emerging commercial products)	TRL 9 (fully commercialized, industrial-scale deployment)
Regulatory Status	CE/FDA approved for molecular diagnostics; limited aquatic pathogen-specific clearances	CE marked for POCT assays; multiple aquatic pathogen test approvals	CE/FDA authorized for biosensor assays; research-stage for aquatic use	CE marked for rapid diagnostic tests; widely approved for field use	CE marked for mobile diagnostic devices; emerging aquatic-specific clearances	Not applicable (environmental monitoring equipment, no diagnostic regulatory requirement)
Key Trade-off	Automation vs. equipment cost	Low cost vs. limited multiplexing	Sensitivity vs. electrode fouling	Simplicity vs. lower sensitivity	Connectivity vs. standardization	Coverage vs. maintenance burden

Note: LOD values represent typical ranges from recent literature (2020–2025). Actual performance varies with target organism, sample matrix, and assay optimization. Bacterial LODs are expressed as CFU/mL; toxin and small-molecule LODs are expressed in molar or mass concentrations as reported.

The platforms surveyed in this section illustrate substantial progress toward field-deployable detection of aquatic microorganisms. Microfluidic LOC systems now enable automated sample-to-answer workflows for complex water matrices, while aptamer-based biosensors provide stable, regenerable recognition elements suitable for continuous monitoring. Smartphone integration has democratized quantitative diagnostics by converting ubiquitous mobile devices into analytical instruments with cloud connectivity. Nevertheless, challenges remain in mitigating sample matrix interference, achieving regulatory standardization, and reducing costs for large-scale aquaculture deployment. Looking forward, the data streams generated by these biosensor platforms can be substantially enhanced through integration with advanced computational approaches. The following section examines how next-generation sequencing and artificial intelligence are further transforming aquatic pathogen surveillance by enabling comprehensive genomic characterization and predictive modeling.

## 5. Next-Generation Sequencing and Artificial Intelligence

The targeted molecular and biosensor-based approaches discussed in the preceding sections excel at detecting known pathogens with high sensitivity, yet they require prior knowledge of the target organism. In contrast, next-generation sequencing (NGS) enables comprehensive, culture-independent characterization of aquatic microbiomes, supporting simultaneous detection of known and emerging pathogens, antimicrobial resistance determinants, and functional traits. Metagenomic and amplicon-based surveys are now being complemented by artificial intelligence (AI) methods that can mine high-dimensional sequence and environmental data for patterns linked to disease risk and water quality. This section examines NGS- and AI-enabled strategies for integrated, predictive surveillance of aquatic microorganisms.

### 5.1. Next-Generation Sequencing and Metagenomic Approaches

Metagenomics, the culture-independent sequencing of all genetic material in a sample, enables unbiased characterization of aquatic microbial communities. Short-read Illumina platforms provide highly accurate, high-throughput profiling of 16S rRNA gene amplicons and shotgun metagenomes, whereas Oxford Nanopore and PacBio long-read technologies offer improved genome assembly and portable field deployment [165,166]. These approaches enable simultaneous detection of bacterial, viral, and eukaryotic pathogens alongside antimicrobial resistance genes in aquaculture environments.

Recent studies demonstrate the utility of NGS for aquaculture surveillance. Rajonhson et al. [165] applied integrative short- and full-length 16S rRNA sequencing to Pacific white shrimp ponds, revealing that ecological niche (intestine, water, sediment) explained the majority of bacterial community variation, with long reads improving species-level resolution for health-relevant taxa. Orin et al. [166] characterized environmental and host-associated microbiota in shrimp aquaculture, identifying functional and taxonomic signatures associated with pond conditions. Gao et al. [167] combined 16S rRNA amplicon and metagenomic sequencing to profile seafood microbiomes, detecting diverse pathogens including *Vibrio* species alongside antimicrobial resistance genes, highlighting seafood as a reservoir for resistance dissemination.

Despite these advances, NGS workflows face constraints including cost per sample, library preparation logistics, and bioinformatics expertise, with turnaround times exceeding those of PCR or LAMP. Portable nanopore sequencing (Oxford Nanopore Technologies) deserves particular attention for aquatic pathogen identification. MinION-based workflows enable real-time analysis of amplicon or metagenomic libraries in the field, with read generation beginning within minutes of library loading. For aquaculture applications, nanopore sequencing has been explored for rapid species-level identification of bacterial pathogens, viral genome characterization, and antimicrobial resistance gene profiling directly from pond water or tissue samples. However, higher per-read error rates compared to Illumina platforms necessitate either sufficient coverage depth or consensus correction algorithms for reliable variant calling. Critical assessment of NGS applications in aquatic diagnostics reveals several limitations specific to this domain. Environmental DNA in aquaculture water degrades rapidly due to UV exposure, microbial nucleases, and variable pH/salinity, complicating library preparation from field samples. Humic substances and other PCR inhibitors that affect amplification-based methods also interfere with library preparation enzymes. Bioinformatic challenges are compounded by the limited representation of aquatic pathogen genomes in reference databases, particularly for emerging viruses and parasites. Targeted sequencing panels, such as amplicon-based approaches for aquaculture-relevant pathogen panels, offer a pragmatic bridge between the breadth of untargeted metagenomics and the specificity of single-target PCR, enabling simultaneous monitoring of dozens of priority pathogens at reduced cost and computational complexity. As datasets grow in complexity, AI and machine learning are increasingly essential to distill NGS outputs into actionable indicators for disease prediction.

### 5.2. Artificial Intelligence and Machine Learning Applications

Artificial intelligence (AI) and machine learning (ML) increasingly support aquatic diagnostics by extracting patterns from complex, multivariate data streams. Supervised learning enables classification of diseased versus healthy fish and prediction of water quality parameters, while unsupervised clustering reveals latent health states. Common models include Random Forests, support vector machines, convolutional neural networks (CNN), and recurrent architectures such as LSTM [164,168,169,170,171]. These models leverage image, sensor, and hyperspectral data to enable predictive early warning systems.

Deep learning-based computer vision has been explored for image-based fish disease diagnosis. Rao [168] developed a Faster R-CNN system trained on annotated images of diseased and healthy fish, achieving 98% detection accuracy for automated lesion-based diagnosis. Biswas et al. [170] applied transfer learning with VGG-16, MobileNetV2, and Inception-V3 combined with SVM classification, demonstrating improved sensitivity and specificity compared with single-model baselines for robust disease recognition across multiple conditions.

AI/ML applications directly integrated with molecular detection workflows represent a rapidly growing area. Automated interpretation of LAMP and RPA results from colorimetric or fluorescent images using convolutional neural networks has been demonstrated to reduce subjective operator bias and enable quantitative readout from qualitative assays. Machine learning-assisted primer and crRNA design tools (e.g., using sequence feature extraction and binding energy prediction) have been developed to optimize CRISPR diagnostic assay design, reducing the experimental iteration required for new target development. In metagenomic analysis, deep learning classifiers trained on sequence composition features enable rapid taxonomic assignment and pathogen identification from complex environmental samples, complementing traditional alignment-based approaches. Predictive models that integrate molecular detection data (e.g., qPCR-derived pathogen loads) with environmental parameters (temperature, dissolved oxygen, stocking density) for disease risk forecasting represent a particularly promising application, though such models require extensive validation across diverse aquaculture systems before deployment.

Despite these advances, AI deployment in aquaculture remains constrained by limited training datasets, domain shifts across farms, and black-box decision processes. Model generalizability and external validation across species and culture systems are critical prerequisites before AI tools can be reliably integrated with NGS and biosensor platforms for routine aquatic health management.

### 5.3. Integration and Future Perspectives

The convergence of NGS, AI/ML, and biosensor platforms is enabling multi-layered surveillance systems that couple comprehensive genomic profiling with continuous environmental monitoring. Emerging approaches apply AI to interpret metagenomic outputs, link microbial signatures to disease risk, and optimize biosensor networks, while smartphone-embedded deep learning extends diagnostic capabilities to resource-limited settings. Looking ahead, the combination of autonomous sampling, on-site sequencing, dense sensor networks, and AI-driven analytics points toward self-optimizing, predictive health management in aquaculture. Realizing this vision will require interoperable data standards, explainable models, and rigorous field validation across diverse production systems.

## 6. Discussion

### 6.1. Comparative Analysis and Technology Selection

The rapid detection technologies reviewed in this article span a broad spectrum of complexity, cost, and deployment readiness, each offering distinct advantages for specific aquaculture and environmental monitoring scenarios. Nucleic acid amplification methods-PCR, LAMP, and RPA-remain the backbone of molecular diagnostics, with qPCR providing the gold standard for quantification and regulatory compliance, while isothermal methods (LAMP, RPA) enable equipment-minimal field deployment with detection limits typically in the range of 1–100 copies per reaction within 20–60 min. CRISPR-Cas systems have emerged as powerful signal amplification modules that, when coupled with isothermal preamplification, achieve attomolar sensitivity with visual or fluorescent readouts suitable for point-of-care applications.

Biosensor and microfluidic platforms address the critical need for automation and user-independent operation. Electrochemical aptasensors achieve high sensitivity for detecting toxins and small molecules, whereas colorimetric and smartphone-integrated systems prioritize accessibility and connectivity over ultrahigh detection performance. Microfluidic lab-on-a-chip devices are increasingly capable of true sample-to-answer workflows, yet cost and manufacturing scalability hinder their widespread application. At the system level, NGS enables unrivaled breadth in pathogen discovery and resistome profiling, while AI/ML models mine complex environmental and genomic datasets to extract predictive insights.

Technology selection should be guided by the specific use case: routine farm-level screening may favor rapid, low-cost isothermal or biosensor methods; regulatory testing and outbreak investigation demand the quantitative rigor of qPCR or digital PCR; and comprehensive surveillance of emerging threats benefits from metagenomic approaches augmented by machine learning analytics. To facilitate practical decision-making, the key trade-offs summarized in Table 3 can be mapped to specific aquaculture use scenarios:

Routine farm-level screening (daily/weekly health checks): Prioritize rapid turnaround (<30 min), visual readout, minimal equipment, and low per-test cost. Recommended: colorimetric LAMP, RPA-LFD. Key trade-off: Limited multiplexing and semi-quantitative results.

Point-of-care outbreak investigation (emergency response to mortality events): Prioritize high sensitivity, specificity, and rapid turnaround with portable equipment. Recommended: RPA-CRISPR/Cas12a with fluorescent or LFD readout; portable qPCR. Key trade-off: Higher reagent cost and cold-chain dependence vs. colorimetric LAMP.

Regulatory testing and health certification (export, import, zonal certification): Prioritize quantitative accuracy, established validation, and regulatory acceptance. Recommended: qPCR (OIE-listed assays), dPCR for reference quantification. Key trade-off: Requires laboratory infrastructure, trained personnel, and longer turnaround.

Continuous environmental monitoring (water quality surveillance): Prioritize continuous or semi-continuous operation, remote data transmission, and long-term stability. Recommended: IoT-enabled electrochemical biosensors for indicator parameters; periodic molecular testing triggered by sensor alerts. Key trade-off: Current biosensors target physicochemical parameters; pathogen-specific continuous biosensors remain limited.

Comprehensive pathogen and resistome surveillance (research, regulatory monitoring): Prioritize breadth of detection, novel pathogen discovery, and AMR profiling. Recommended: Metagenomic NGS with AI-assisted analysis. Key trade-off: Highest cost, longest turnaround, requires bioinformatics expertise.

For routine screening on farms where cost and simplicity are of paramount importance, colorimetric LAMP or RPA-LFD assays are preferred despite their limited multiplexing capability, as they require minimal training and no specialized equipment (Table 3, “Low cost vs. limited multiplexing”). When quantitative accuracy and regulatory compliance are required, qPCR or dPCR remain the methods of choice, accepting higher equipment and personnel costs. For point-of-care outbreak investigation requiring both speed and specificity, CRISPR-Cas12a coupled with RPA offers attomolar sensitivity with visual readout within 30–60 min. At the systems level, microfluidic LOC platforms provide the highest degree of automation, but manufacturing scalability and per-device costs remain barriers. Finally, for comprehensive surveillance, metagenomic NGS provides unparalleled breadth, with the trade-off of higher costs and dependence on bioinformatics expertise.

### 6.2. Current Challenges

Despite substantial progress, several challenges impede the translation of these technologies from research settings to routine aquaculture practice. Sample matrix complexity remains a universal obstacle: aquaculture water, sediments, and biological tissues contain PCR inhibitors, particulate matter, and competing nucleic acids that compromise assay sensitivity and reproducibility. While on-chip sample preparation and magnetic bead-based extraction have improved robustness, performance validation across diverse water types and species remains limited.

Standardization and regulatory acceptance present additional hurdles. The lack of harmonized protocols, reference materials, and proficiency testing schemes complicates comparison across studies and hinders regulatory adoption of novel methods. Many promising biosensor and CRISPR-based assays have been validated only with spiked samples or limited field trials, leaving questions about real-world performance unanswered. Cost and infrastructure constraints are particularly acute in low- and middle-income aquaculture regions, where the majority of global production occurs. Although isothermal and paper-based platforms reduce equipment requirements, reagent costs, cold-chain logistics, and technical training remain significant barriers. For NGS and AI-based approaches, bioinformatics expertise and computational infrastructure add further layers of complexity. Finally, data integration and interoperability challenges emerge as monitoring systems become more sophisticated. Combining real-time sensor data, molecular diagnostics, and genomic surveillance into unified decision-support platforms requires standardized data formats, secure cloud architectures, and validated predictive models—capabilities that remain nascent in most aquaculture operations.

Quality assurance and quality control (QA/QC). The absence of standardized positive and negative controls, certified reference materials, and proficiency testing programs for most emerging aquatic pathogen detection platforms severely limits the comparability of results across laboratories and studies. While qPCR benefits from established QA/QC frameworks (e.g., MIQE guidelines), no equivalent standards exist for LAMP, RPA, CRISPR-based, or biosensor assays in aquatic diagnostics.

Cross-laboratory reproducibility. Most platforms reviewed here have been validated only in the originating laboratory, with very few multi-center studies. Differences in operator skill, environmental conditions, sample handling, and equipment calibration can significantly affect assay performance, yet inter-laboratory ring trials remain rare for emerging detection technologies.

Cold-chain and reagent stability. Many molecular assays depend on enzymes (Bst polymerase, recombinase, Cas proteins) and reporter molecules that require −20 °C storage and are sensitive to freeze–thaw cycles. While lyophilized reagent formats for RPA and some LAMP assays have demonstrated stability at ambient temperatures for weeks to months, the long-term stability of CRISPR-Cas reagents under tropical field conditions—where most global aquaculture production occurs—remains poorly characterized.

Operator training and skill requirements. True point-of-care deployment requires minimal operator expertise. While colorimetric LAMP and RPA-LFD assays approach this goal, many CRISPR-based and microfluidic platforms still require pipetting skills, knowledge of contamination control procedures, and the ability to interpret ambiguous results. Standardized training curricula and pictorial standard operating procedures are needed to support technology transfer to farm-level personnel.

Contamination control. Isothermal amplification methods, particularly LAMP, generate extremely high amplicon concentrations (up to 10^9^ copies) that pose significant carryover contamination risks. Closed-tube formats, physical separation of pre- and post-amplification areas, and UDG/dUTP systems have been proposed but are not universally adopted. For CRISPR-based assays, the two-step format introduces tube-opening transfer steps that increase contamination risk relative to one-pot alternatives.

Economic feasibility. While per-assay reagent costs for LAMP and RPA are substantially lower than for NGS, the total cost of implementation must include equipment purchase/maintenance, quality control materials, sample preparation consumables, operator training, and waste disposal. A comprehensive cost-effectiveness analysis comparing these technologies for specific aquaculture surveillance scenarios has not been published.

Waste management. Molecular diagnostic assays generate biohazardous waste including used reaction tubes, lateral flow strips, and microfluidic devices potentially contaminated with amplified nucleic acids. Proper disposal protocols are essential to prevent environmental contamination and false positive results from carryover, yet waste management guidelines specific to field-deployed aquaculture diagnostics are lacking.

### 6.3. Sample Preparation: A Critical but Underappreciated Bottleneck

Sample preparation, encompassing sample collection, concentration, lysis, and nucleic acid extraction, represents arguably the most significant bottleneck in translating rapid molecular assays to true point-of-care aquaculture applications. While amplification and detection steps have been dramatically miniaturized and simplified, the upstream sample preparation workflow remains time-consuming, labor-intensive, and often requires laboratory infrastructure.

For aquaculture water samples, the challenge is two-fold: target organisms are typically present at very low concentrations (requiring large-volume concentration by filtration, flocculation, or ultracentrifugation) and the matrix contains diverse PCR/LAMP inhibitors (humic substances, organic particulates, salts, heavy metals) that must be removed prior to amplification. Current approaches include membrane filtration with bead-beating lysis, magnetic bead-based nucleic acid capture, chemical flocculation (e.g., Fe-based methods), and simplified heat-lysis protocols. However, there is no universally optimized sample preparation workflow that is simultaneously rapid, field-compatible, and effective across the diversity of aquatic matrices encountered in global aquaculture. This bottleneck is reflected in the discrepancy between reported assay turnaround times and actual sample-to-answer times. For example, while a LAMP or RPA reaction may be completed in 20–30 min, the total time from raw sample to result, including concentration, extraction, and detection, typically exceeds 60–90 min, narrowing the practical speed advantage over qPCR. Future development should prioritize integrated sample preparation modules, extraction-free approaches (e.g., direct heat lysis of fish mucus or gill swabs), and microfluidic platforms with on-chip sample processing to achieve true point-of-care performance.

### 6.4. Future Opportunities

The convergence of the technologies reviewed here points toward increasingly integrated, autonomous monitoring systems. Miniaturized, multiplexed biosensors embedded in IoT networks could provide continuous baseline surveillance, triggering targeted molecular or metagenomic testing when anomalies are detected. Advances in portable nanopore sequencing and edge-computing AI models are progressively lowering the barriers to on-site genomic analysis, potentially enabling same-day pathogen identification and antimicrobial resistance profiling at farm level.

Commercial translation will require partnerships between technology developers, aquaculture producers, and regulatory agencies to establish validation frameworks, cost-sharing models, and training programs. Open-source assay designs, reagent lyophilization for ambient storage, and smartphone-based data platforms offer pathways to democratize access in resource-limited settings.

Ultimately, the vision of predictive, precision aquaculture health management—where environmental, molecular, and genomic data streams are continuously integrated to anticipate disease outbreaks and optimize interventions—is within technological reach. Realizing this vision will depend not only on continued innovation but also on coordinated efforts to address standardization, validation, and equitable access across the global aquaculture sector.

## 7. Conclusions

This review comprehensively synthesizes the latest advances in rapid detection technologies for aquatic microorganisms from 2020 to 2025, spanning nucleic acid amplification, CRISPR-Cas diagnostics, microfluidic/biosensor platforms, next-generation sequencing, and artificial intelligence applications, and clarifies the evolutionary trajectory of aquatic microbial monitoring from laboratory-dependent, reactive detection to field-deployable, data-driven, and predictive surveillance. It is evident that no single detection technology can fully meet the diverse demands of aquatic microbial monitoring; instead, different technologies exhibit distinct advantages in targeted scenarios. Nucleic acid amplification technologies remain the foundational core, with qPCR and dPCR serving as the gold standard for quantitative accuracy and regulatory compliance in centralized laboratory testing, while isothermal amplification techniques (LAMP, RPA) stand out for on-site rapid screening due to their low instrumentation requirements and fast turnaround times. The integration of CRISPR-Cas systems with isothermal amplification has addressed the long-standing issue of non-specific amplification in traditional molecular assays, achieving ultrahigh sensitivity and specificity at constant temperatures, and emerging as the optimal technical combination for point-of-care aquaculture diagnostics. Microfluidic lab-on-a-chip and biosensor technologies have further realized the automation and miniaturization of detection workflows, with smartphone and IoT integration enabling the connectivity and real-time transmission of diagnostic data, effectively bridging the gap between benchtop research and on-site application. Next-generation sequencing provides an unbiased approach for comprehensive microbiome profiling, pathogen discovery, and antimicrobial resistance gene detection, while artificial intelligence and machine learning extract actionable predictive insights from complex multi-dimensional datasets, laying the foundation for early disease warning in aquaculture systems.

Despite the remarkable technological progress, several core challenges still restrict the large-scale practical application of these rapid detection technologies in aquatic microbial monitoring. Complex aquatic environmental matrices introduce severe inhibitor interference and low pathogen concentration issues, making sample preparation the universal bottleneck for all detection methods; the lack of harmonized protocols, certified reference materials, and proficiency testing schemes hinders the standardization and regulatory acceptance of novel technologies; additionally, high implementation costs, strict cold-chain requirements for reagents, and insufficient technical training resources create significant barriers for technology popularization in resource-limited aquaculture regions. Contamination control of amplification-based assays and long-term stability of detection platforms under field conditions also remain to be further optimized.

Looking ahead, the future development of aquatic microbial rapid detection technologies will center on the integration and integration of multiple technical systems, with the ultimate goal of constructing autonomous, intelligent, and integrated monitoring platforms. Key research directions will include the development of integrated sample-to-answer devices that combine on-chip sample preparation, amplification, and detection, to simplify operational procedures and improve detection efficiency; the optimization of CRISPR-Cas and isothermal amplification coupling systems, to realize one-pot reactions and reduce reagent costs; the integration of pathogen-specific biosensors with IoT networks, to achieve continuous real-time environmental monitoring and trigger targeted molecular testing for abnormal signals; and the deep fusion of portable nanopore sequencing with edge-computing AI models, to lower the threshold for on-site genomic analysis. Moreover, to ensure the equitable access of these advanced technologies to aquaculture operations of all scales worldwide, it is critical to strengthen cross-disciplinary cooperation between researchers, technology developers, aquaculture producers, and regulatory agencies, to establish standardized validation frameworks, cost-sharing mechanisms, and accessible technical training programs, and to promote the development of open-source assay designs and ambient-stable reagent formulations. In summary, the continuous innovation and integration of aquatic microbial detection technologies will provide strong technical support for the sustainable development of the aquaculture industry, the protection of aquatic ecological balance, and the safeguarding of public health, and the vision of predictive and precision aquaculture health management is expected to be gradually realized with the joint efforts of all stakeholders in the field.

## Figures and Tables

**Table 2:** Representative CRISPR-Cas platforms for aquatic microbial detection.

Target	Category	Platform	LOD	Time	Reference
CyHV-2	Fish virus	RAA-Cas12a	10 copies/rxn	60 min	[127]
CyHV-3	Fish virus	RAA-SHERLOCK-LFD	100 ag/µL	<60 min	[134]
NNV	Fish virus	Dual-Cas12a RT-RAA	0.5 copies/µL	30 min	[135]
*V. parahaemolyticus*	Bacterium	RPA-Cas12a E-CRISPR	32 CFU/mL	<60 min	[128]
*Y. ruckeri*	Bacterium	Cas13a SHERLOCK	qPCR-level	<90 min	[130]
mcyE gene	Cyanotoxin	RPA-Cas12a	48 copies/µL	50 min	[126]
*E. coli* O157:H7	Waterborne	Cas12a-AEFB	176 CFU/mL	—	[129]

## Data Availability

No new data were created or analyzed in this study. Data sharing is not applicable to this article.
